# Closed Rupture of the Extensor Hallucis Longus (EHL) Tendon Due to Forced Traumatic Hyperflexion of the Hallux: A Case Report

**DOI:** 10.7759/cureus.55137

**Published:** 2024-02-28

**Authors:** Georges F Bassil, Fadi Nader, Achraf Lajmi, Zied Missaoui

**Affiliations:** 1 Orthopedic Surgery, Lebanese University Faculty of Medicine, Beirut, LBN; 2 Orthopedics and Trauma, Université Paris Cité, Paris, FRA; 3 Orthopedic Surgery, Grand Hôpital de l'Est Francilien - Site de Meaux, Meaux, FRA

**Keywords:** extensor hallucis longus (ehl), end to end, surgical repair, big toe, delayed presentation, closed rupture

## Abstract

A closed spontaneous rupture of the extensor hallucis longus (EHL) tendon is an infrequent yet challenging clinical occurrence, typically associated with systemic conditions (diabetes mellitus or rheumatoid arthritis). A closed EHL rupture, however, exists but is only reported as scattered cases in the literature. This article presents a unique case of a traumatic EHL tendon rupture in a patient without underlying predisposing factors. A 66-year-old woman, previously healthy, presented with an inability to dorsiflex her big toe following trauma, showcasing the clinical triad of pain, edema, and deficit in big toe extension. Magnetic resonance imaging confirmed a 5.9 cm EHL tendon gap that was treated by primary end-to-end repair of the ruptured tendon. The aim of this case report is to provide an overview of the literature available concerning the classification and treatment of EHL rupture and to assist in the early diagnosis and treatment of this rare condition.

## Introduction

A closed rupture of the extensor hallucis longus (EHL) tendon is infrequent, typically encountered in cases associated with underlying systemic conditions, such as diabetes mellitus or rheumatoid arthritis [1]. While these systemic factors are recognized as predisposing risks, closed EHL ruptures can also result from a variety of other causes. These include post-traumatic ischemic degeneration, which diminishes blood supply, leading to tissue death [2]; attrition over osteophytes, causing gradual wear and tear [1]; iatrogenic injuries following ankle arthroscopy, disrupting tendon architecture [3]; and steroid injections for degenerative joint disease, inducing tissue degeneration [4]. These processes weaken the tendon, increasing susceptibility to rupture, especially during extreme plantarflexion [2] or even spontaneously [1,3,5]. In the event of an EHL injury, numerous investigators have recommended surgical intervention over conservative treatment to prevent functional deficits of the foot [6]. This case report presents an exceptional instance of closed traumatic rupture of the EHL tendon in a patient who lacked any underlying systemic or local predisposing factors. The report further details the classification and surgical technique employed to restore functional hallux extension and normal gait.

## Case presentation

**Figure 1:**
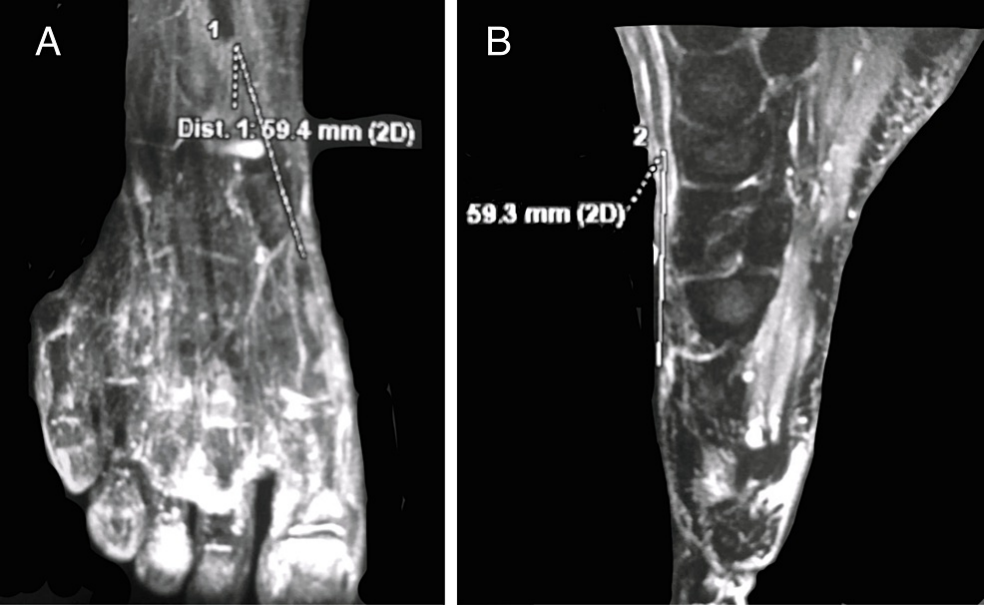
MRI images (A: coronal view; B: sagittal view) showing a 5.9 cm gap between the two ends of the ruptured extensor hallucis longus (EHL) tendon

We present a case of a 66-year-old retired female patient, previously healthy, who presented to the orthopedic clinic with an inability to extend her big toe. The initial injury occurred a month prior when she hit her big toe against the edge of a chair while walking at home. The emergency room physician suspected a fracture, but the X-ray did not show any, and she was discharged with pain management, ice application, and rest. However, the patient developed difficulties in her gait and experienced a deficit in big toe dorsiflexion. Further evaluation through an MRI (Figure 1) revealed a rupture of the EHL tendon at the musculo-tendinous portion, with a 5.9 cm gap between the two ruptured ends.

**Figure 2:**
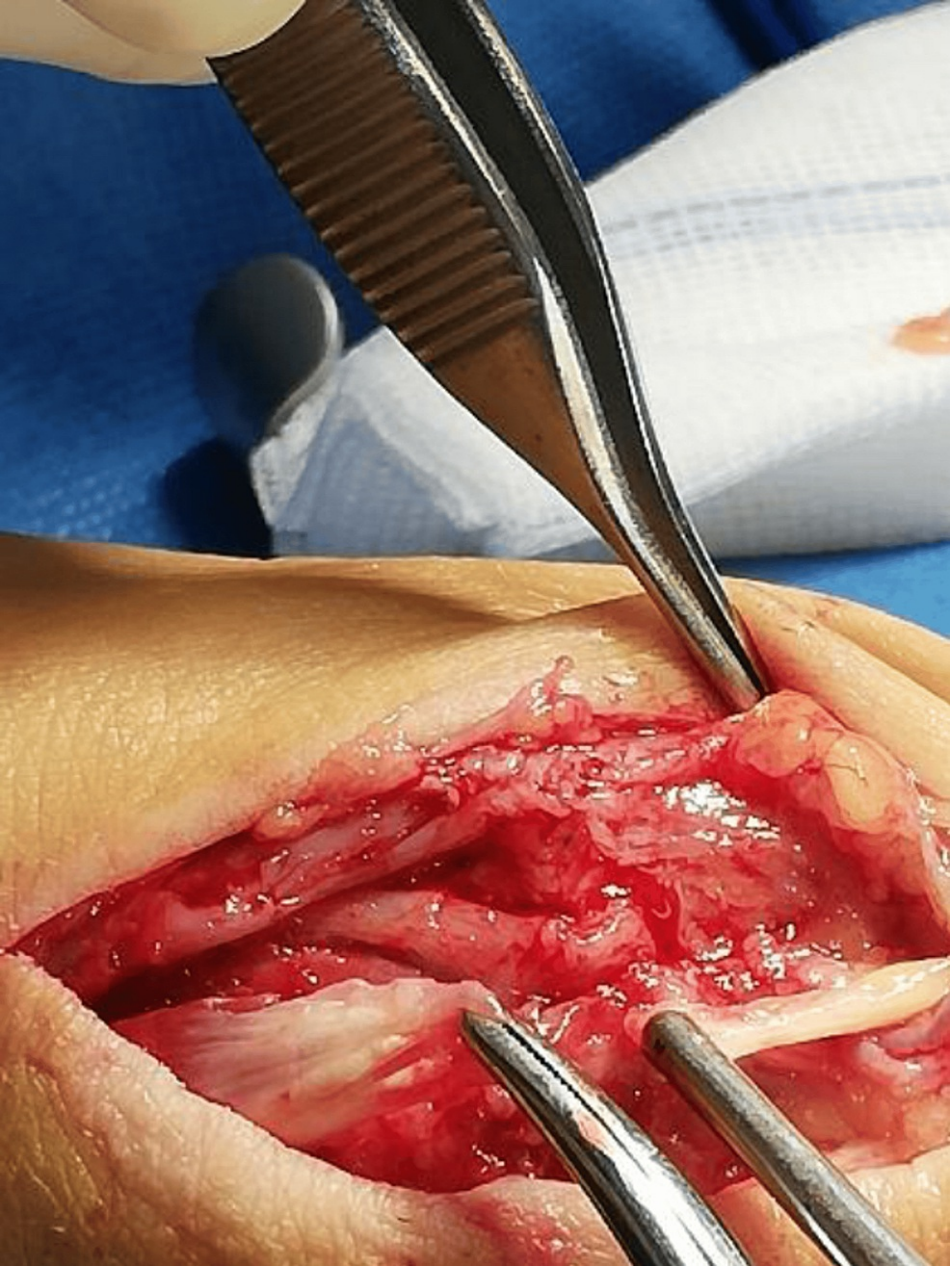
Intraoperative clinical image of the ruptured ends of the extensor hallucis longus (EHL) tendon at its musculotendinous junction

**Figure 3:**
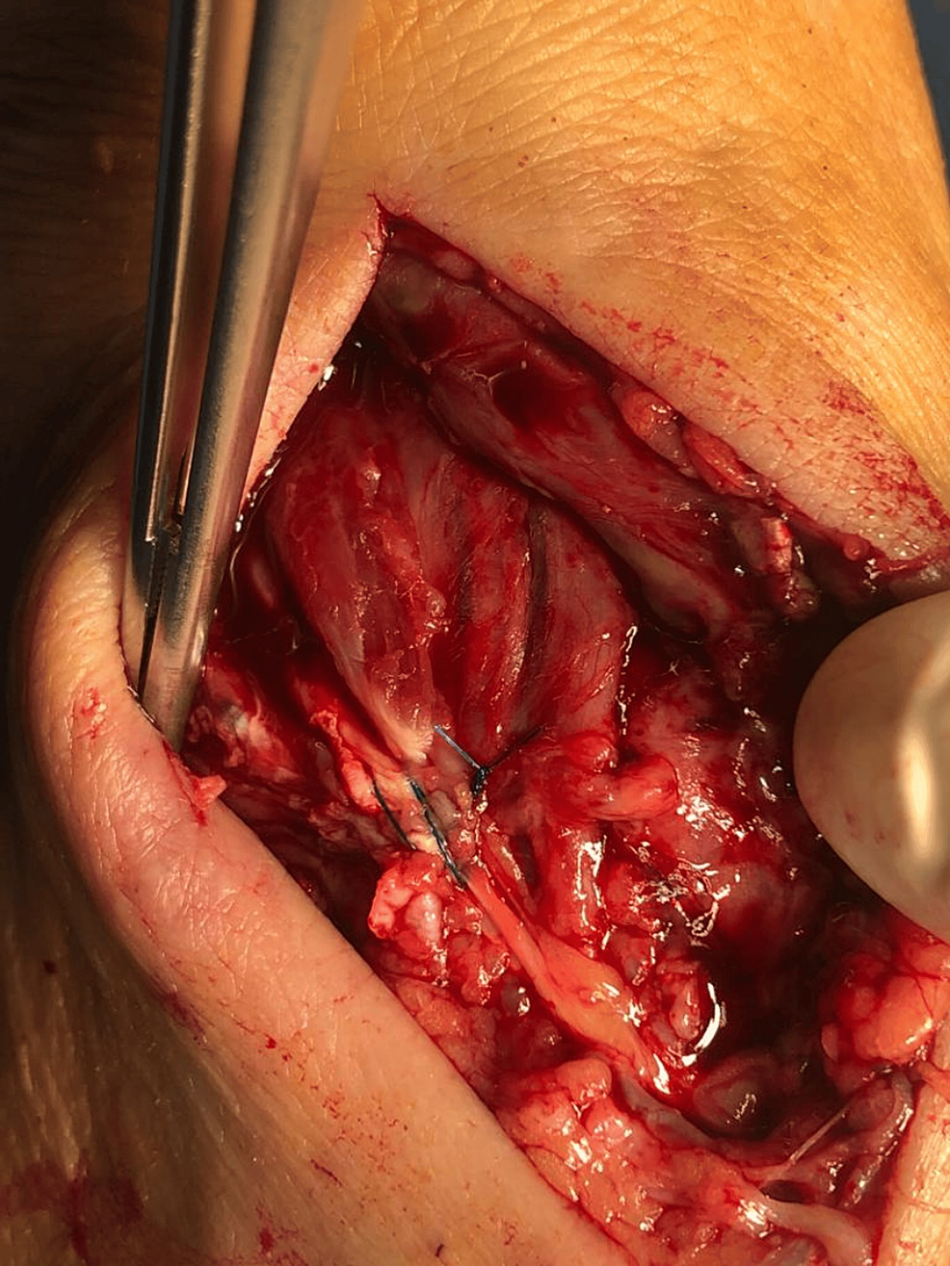
Intraoperative image of the extensor hallucis longus (EHL) tendon after primary end-to-end repair

Following this diagnosis, the patient was referred to the orthopedic clinic, where she was hospitalized and taken to the operating room. A longitudinal incision (Figure 2) was made over the dorsal aspect of the first tarsometatarsal ray from the middle third of the first metatarsal bone going proximally over the navicular bone. Dissection was done to expose the distal tendinous portion and the proximal musculo-tendinous portion of the EHL. No osteophyte was identified during surgical exploration of the tarsal and metatarsal bone adjacent to the path of the ruptured EHL tendon. The primary end-to-end suture of the EHL longus tendon was done by a core stitch using prolene 2-0 and reinforced by running 4-0 vicryl sutures (Figure 3).

**Figure 4:**
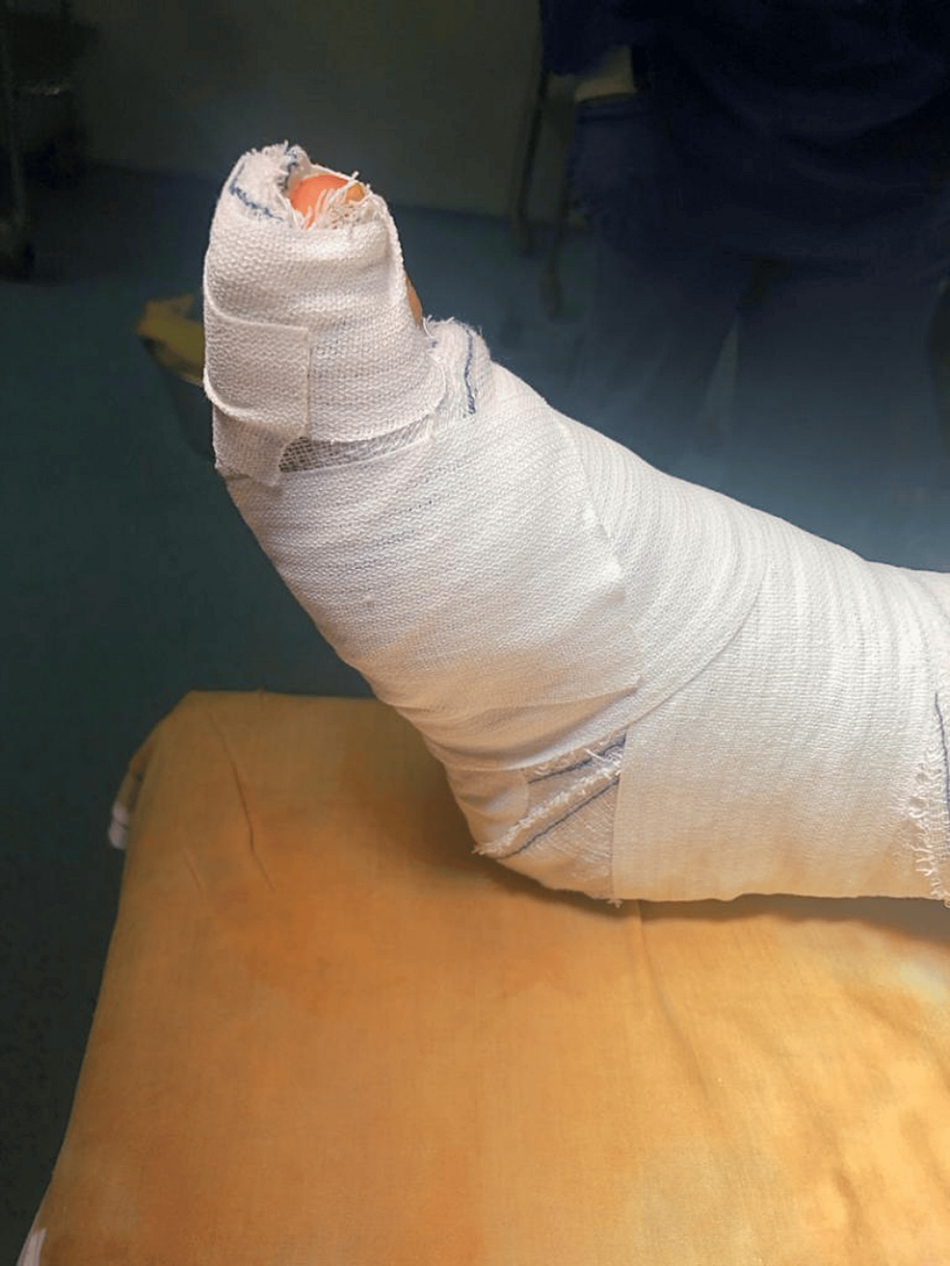
Immobilization of the big toe in dorsiflexion using a posterior splint

**Figure 5:**
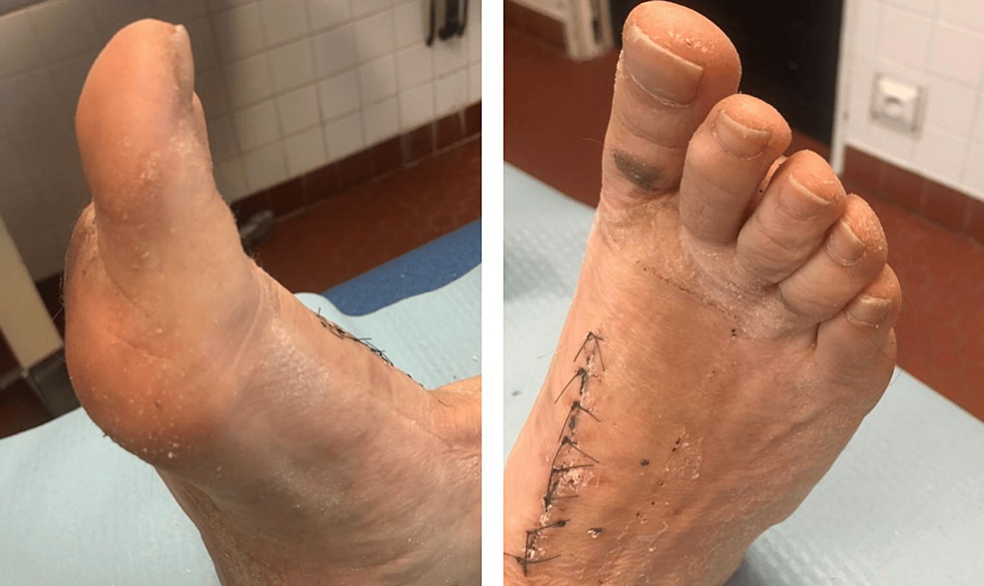
Follow-up images of the wound at two weeks postoperative for stitch removal

Care was taken to repair the EHL in such a way that both the EHL and the EDC (extensor digitorum communis) of the big toe were under tension, and the big toe was in slight dorsiflexion. Immobilization of the big toe in dorsiflexion and the ankle in a neutral position was done using a posterior splint (Figure 4). Skin stitches were removed at two weeks postoperatively, no wound complication was seen, and the big toe's normal position was restored (Figure 5). Non-weight-bearing mobilization was allowed on the same day after the operation with passive dorsiflexion of the big toe as tolerated immediately after the intervention. The immobilization of the big toe was completely removed at six weeks postoperatively, and the patient was started on passive and active physiotherapy sessions to regain strength and mobility of the big toe extensors with progressive weight bearing as tolerated. The patient was able to walk full weight bearing at 10 weeks postoperatively and resumed her normal daily activities. Her final control was at three months postoperatively with no complications and preserved function of the EHL.

## Discussion

**Figure 6:**
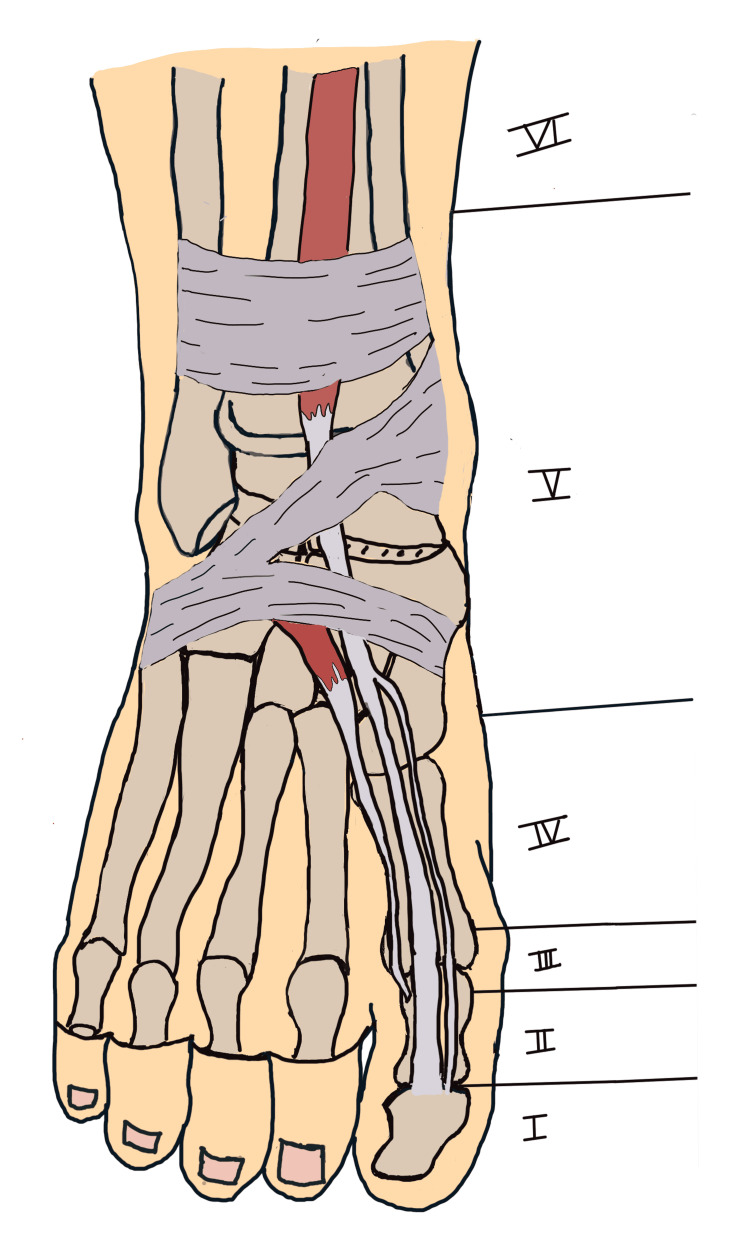
Topographic division of the EHL into six zones Reference: Al-Qattan [7] (Image credits: Dr. Georges Bassil)

A closed spontaneous rupture of the EHL tendon presents a unique challenge due to its infrequency. Several mechanisms have been proposed for such injuries, including repetitive microtrauma [5], rupture secondary to talar neck osteophytes [1], and decreased blood supply to the EHL tendon following a previous fracture of the tibia or fibula [2]. A patient with an EHL rupture will present with the toe in slight plantar flexion and weakness or a complete deficit in big toe dorsiflexion. We should always start with plain radiography of the foot to eliminate associated fractures and to check for the presence of osteophytes that could be the underlying cause of the tendon rupture. Then, we can order an ultrasound or MRI to confirm the EHL rupture and to measure the gap between the proximal and distal ends of the tendon. The EHL tendon traverses three critical "tunnels" - the superior and inferior extensor retinaculum and, distally, the extensor hood apparatus. Based on these anatomical structures, Al-Qattan divided this tendon into six topographic zones (Figure 6), with the most proximal located at the ankle just proximal to the superior extensor retinaculum and the most distal is located at the EHL insertion [7].

In our case, the rupture of the EHL tendon was in zone IV, which is a zone located between the metatarsophalangeal joint and the inferior extensor retinaculum of the foot. Various surgical interventions have been employed for the repair of EHL tendon injuries, including end-to-end primary repair [8], peroneus tertius tendon transfer [2], side-to-side tenodesis to the extensor digitorum communis (EDC) tendon [1], division of the EDC to the second toe [6], or the use of bridging materials like fascia lata [9] or semitendinosus tendon grafts [10], and there are even some reports about using residual scar tissues as a graft [11] in cases of neglected EHL rupture. It seems that there is a consensus about end-to-end repair of the acute EHL rupture because neglecting the repair could lead to the development of various great toe afflictions, such as hammered hallux [12], dorsal bunion [13], hallux flexus [14], hallux malleus [12], and malperforans ulceration [15], and will affect the normal gate of the patient. Furthermore, delayed presentation will result in tendon retraction, and trimming of the tendon ends will leave a gap requiring a tendon transfer or graft. It is also crucial to maintain the adequate tendon tension necessary for the normal function of the EHL muscle.

A list of the published cases about closed EHL ruptures is illustrated in Table 1. 

**Table 1:** List of closed EHL rupture publications similar to our case report, including the zone of laceration and treatment done for each case EHL: extensor hallucis longus

Authors	Case presentation	Zone of laceration	Treatment
Fadel et al. (2008) [1]	Closed rupture of the EHL tendon due to a talar neck osteophyte	Zone V	Removal of the talar osteophytes, and because end-to-end suturing was not possible, suturing of both tendon ends to the EDL tendon was done using Ethibond sutures.
Sung Hon Won et al. (2023) [11]	A neglected closed EHL rupture caused by arthritic adhesions	Zone III	Primary end-to-end repair of the tendon.
Shah et al. (2010) [16]	Closed traumatic rupture of the EHL	Zone III	Primary end-to-end suturing of the ruptured tendon.
Tadros et al. (2013) [17]	Closed traumatic rupture of the EHL	Zone IV	Excision of the ruptured region at the musculocutaneous junction and suturing of the EHL to the main EDC suing polypropylene stitch.
Woo Jong Kim et al. (2021) [18]	Reconstruction of a neglected EHL rupture using scar tissue	Zone IV	Suturing of the EHL scar tissue at the distal tendon end to the proximal EHL tendon stump.

In the present case, we were fortunate to be able to do primary end-to-end suturing of the tendon and to restore its optimal tension. Postoperative care following EHL tendon rupture management is crucial and involves immobilization for approximately six weeks. Excellent results have been obtained with immobilization in a short leg cast with the great toe extended for short periods of time (three to eight weeks) before initiation of physical therapy. Dynamic splinting has been used in one case following repair of a lacerated EHL tendon, and it resulted in an excellent outcome. In our case, the patient was immobilized with a posterior splint that extended into the big toe and maintained the big toe and the foot in extension while allowing passive dorsiflexion of the big toe immediately compared to another case report [4] that used a short leg walking cast with cutting of the area over the dorsum of the toes at two weeks to allow for passive extension exercises. We began passive and active ranges of motion of the big toe with weight bearing as tolerated using regular shoes starting at six weeks compared to a similar case report [4] in which at six weeks, it advanced to a wooden-soled shoe to facilitate transition to a regular shoe, also with weight bearing as tolerated.

## Conclusions

A closed spontaneous rupture of the EHL tendon, although rare, presents a distinct clinical challenge. While systemic conditions often contribute to such ruptures, traumatic cases without underlying factors can result in a complete rupture. Early diagnosis, appropriate surgical repair, and meticulous postoperative care, including immobilization and physiotherapy, contribute to successful outcomes. Satisfactory results are often achieved with end-to-end stitching of the tendon. However, the treatment of neglected EHL rupture remains a subject of controversy, and further studies are needed to provide clear indications and treatment options that suit each case.
